# An Algorithm for Incorporating Economic Evidence Into Clinical Practice Guidelines: Application to the Spanish Guideline for the Management of Chronic Primary Pain

**DOI:** 10.1002/gin2.70045

**Published:** 2025-09-24

**Authors:** Celia Muñoz, Patricia Gavín‐Benavent, Silvia Moler‐Zapata, Lucía Prieto Remón, María Bono Vega, Soledad Isern de Val, on behalf of GuíaSalud's Working Group on Incorporating Economic Evidence into CPGs

**Affiliations:** ^1^ Instituto Aragonés de Ciencias de la Salud Zaragoza Aragón Spain

**Keywords:** algorithm, chronic primary pain, clinical practice guideline, economic evidence

## Abstract

The importance of incorporating economic evidence (EE) such as resource use, cost or cost‐effectiveness evidence into Clinical Practice Guidelines (CPG) is being increasingly recognised by decision makers, including healthcare providers and policy makers. Yet, this is a complex and resource‐intensive process. EE can influence recommendations formulated about interventions in CPGs, especially when the desirable and undesirable effects are balanced. A group of methodologists, including health economists, in GuíaSalud (the organisation of the Spanish National Health System (NHS) that coordinates the national CPG Programme), has made important advances in the development of methodological guidance for how to develop evidence‐based recommendations. In this article, we present an algorithm for informing decisions about incorporating EE in CPGs. The algorithm has three stages: (1) prioritise clinical questions in the CPG according to the influence that EE is expected to have on recommendations; (2) obtain EE for clinical questions that have been prioritised via a systematic review and/or a *de novo* economic evaluation; and (3) use EE to inform recommendations in CPG. We show how the algorithm was applied in the development of GuíaSalud's CPG for the management of chronic primary pain. In doing so, we provide specific guidance on how the algorithm could be applied using concrete examples. We show how this algorithm helps to make the process of incorporating EE into CPGs agile, dynamic and reproducible.

## Summary

- The GRADE approach acknowledges the importance of incorporating economic evidence into clinical practice guidelines (CPGs), but there is still little guidance for how to adequately do so.
- We developed an algorithm to facilitate decision‐making regarding the integration of economic guidance into CPGs.
- We applied the algorithm to the Spanish Guideline for the Management of Chronic Primary Pain, which helped inform some of its recommendations.
- It is necessary to continue advancing towards clearer guidance, including better use of real‐world data, and building capacity to ensure economic evidence is effectively integrated into CPGs.

## 1 Introduction

Clinical practice guidelines (CPGs) are a set of recommendations based on the systematic review (SR) of evidence on the benefit‐risk balance of interventions or technologies. CPGs are developed with the aim of supporting decision‐making by healthcare providers and improving patient care, more generally. Traditionally, CPGs have based their recommendations on clinical factors such as effectiveness, safety, or diagnostic reliability. However, the need for and the relevance of incorporating elements related to efficiency or cost‐effectiveness is being increasingly recognised by decision makers. For an intervention to be considered efficient, it should not only be able to demonstrate to be sufficiently effective and safe, but also that it represents good value for money. That is, the health gains it yields should be greater than the health gains that would be obtained if the resources required for its provision were to be used to deliver other interventions across the system [1, 2].

With the aim of improving the quality of patient care across the Spanish National Health System (NHS), GuíaSalud (https://portal.guiasalud.es/) was founded in 2002 to coordinate the national CPG Programme. Its secretariat is held by the Aragon Health Sciences Institute (*Instituto Aragonés de Ciencias de la Salud*, IACS), which is one of the eight Health Technology Assessment (HTA) agencies conforming the Spanish Network of Agencies for Assessing National Health System Technologies and Performance (*Red Española de Agencias de Evaluación de Tecnologías Sanitarias y Prestaciones del Sistema Nacional de Salud*, RedETS) [3, 4]. These agencies are responsible for the development of CPGs within GuíaSalud and they also collaborate to develop other evidence‐based products such as HTA reports. All CPGs developed within GuíaSalud are developed using the GRADE system (Grades of Recommendation Assessment, Development and Evaluation) [5]. The GRADE system is a tool for evaluating the quality of evidence and grading the strength of recommendations in the context of CPG development [5, 6, 7]. GRADE offers a transparent and structured process for developing and presenting evidence summaries and for formulating recommendations. One of its main advantages is that it shows a clear separation between the quality of evidence and the strength of the recommendations, allowing for an explicit evaluation of the importance of the outcomes of the different management strategies [5, 8].

The formulation of recommendations using GRADE should consider not only the benefit‐risk balance, but also factors such as equity and feasibility, as well as economic criteria such as the resource use, costs or cost‐effectiveness of the intervention [9, 10]. Incorporating economic evidence (EE) in CPGs is a complex process, both due to difficulties in the quantification of costs and health effects, and to the differences that may exist between contexts where evidence is generated and those where the CPG is developed. As part of the process of incorporating EE in CPGs, additional considerations such as the perspective for evaluating effects and costs should be evaluated and adequately justified in the early stages of its development (e.g., in the Spanish context, the perspective of the Spanish NHS should be favoured, expanding in specific cases towards a broader perspective [11]).

The lack of exemplar applications and methodological guidance for incorporating EE in the development of CPGs has resulted in high variability in practice. For this reason, in 2019, a working group (WG) of methodologists was established within GuíaSalud and RedETS with the mission of establishing a series of quality standards and guidance for how EE should be incorporated in CPGs. The creation of this WG highlighted the interest in supporting the exchange of information and experiences in CPGs and other evidence‐based health technology evaluations [3]. As part of its efforts, the WG developed an algorithm for guiding decision‐making in the process of incorporating EE in the formulation of recommendations in CPGs. The algorithm was developed to help address the challenges this process entails, and help target resources to those areas where EE would be most needed. The CPG for the management of chronic primary pain (CPP CPG) was commissioned to IACS, and was the first CPG to apply the algorithm, beginning its work in 2021. The aim of this article is to present the algorithm and methods used to develop it (Sections 2 and 3), and how it was applied in the development of the CPP CPG (Section 4). It also discusses the main strengths and limitations of the work done, as well as some areas for future work (Section 5).

## 2 Methods for Developing an Algorithm for Incorporating EE Into CPGs

### 2.1 Background

The WG on Incorporating Economic Evidence in CPGs (EE‐WG) was established within GuíaSalud with the goal of developing methodological consensus, guiding and standardising the process of incorporating EE in the development of CPGs in the Spanish context. Individuals with experience in developing CPGs in GuíaSalud and who had different technical profiles, including health economists and methodologists are part of this WG. Through regular meetings and new internal communication channels, the EE‐WG promotes collaborative work, and facilitates the exchange of experiences, as well as the development of good practice and methodological guidelines. Over the years, the EE‐WG has reached agreements on several methodological issues. One key achievement has been the development of a decision‐making algorithm that facilitates the inclusion of EE in CPGs. The development of the algorithm followed an iterative, consensus‐based process, articulated in four main phases, which are described below in turn.

### 2.2 Experiences Incorporating EE Into CPGs in Guíasalud

In the initial phase, members of the EE‐WG discussed previous experiences on incorporating EE into CPGs. These included CPGs on paediatric palliative care, generalised anxiety disorder in primary care, prostate cancer and type‐2 diabetes mellitus, among other clinical areas. These CPGs provided learning experiences that were shared and discussed with the group. Common methodological challenges were also identified, including the absence of clear criteria for prioritising clinical questions from an economic perspective, difficulties in extracting relevant data on resource use in clinical trials, variability in the quality and applicability of published economic evaluations, and the need to adapt economic evidence profiles to the national context. Different approaches to presenting EE were also discussed, including the estimation of unit costs and the use of summary tables. These experiences allowed the group to better delineate the scope of the algorithm, emphasising the need for it to be a flexible tool, adaptable to different clinical and thematic contexts, and compatible with the capacities and resources available for the development of CPGs.

### 2.3 Literature Review of Tools and Guidance

The EE‐WG conducted a review of international methodological manuals, tools and guidelines to identify different approaches to incorporating EE into CPGs. Decision‐making frameworks and strategies such as NICE's [12] ‘Economic Plan’ were identified, as well as schemes used by the Australian National Health and Medical Research Council, the Colombian Ministry of Health CPG manual and the US Preventive Services Task Force. Since no existing methodology could be directly adopted, it was deemed necessary to design a new, tailor‐made approach. Previous methodological guidance identified served as a starting point for our own development.

### 2.4 An Iterative Approach for Co‐Creating the Algorithm

A first version of the algorithm was developed and refined through multiple meetings of the EE‐WG. In these meetings, necessary adjustments were discussed to adapt it to the context of the GuíaSalud CPG Programme, such as the incorporation of prioritisation tools, templates for EE profiles or flexible review strategies. Methodological templates were developed, such as outlines for EE synthesis and adapted evidence profile formats, based on tools previously used by RedETS. The entire process was documented through minutes and working materials, ensuring traceability and transparency in decision‐making.

### 2.5 External Consultation and Validation

The EE‐WG shared the different versions of the algorithm in national and international fora. To align the work with international standards, the EE‐WG was in continuous contact with the GRADE group on economic evidence. This study towards external validation helped to reinforce the robustness and applicability of the algorithm. As a result, a dynamic, adaptable and reproducible algorithm was obtained for the incorporation of economic evidence in the NHS CPGs. We discussed its main stages and structure in the following sections (Section 3), as well as a case study of how it was applied in one of GuíaSalud's CPGs (Section 4).

## 3 Algorithm for Incorporating EE Into CPG

### 3.1 Some Preliminary Considerations for Successful Integration of Economic Evidence in CPGs

The starting point for any CPG is to define its scope and objectives and select the members of the Guideline Development Group (GDG). The GDG is made up of methodologists, healthcare professionals, patients and/or caregivers or representatives of patients' associations and policy makers. The presence of a health economist is critical to ensure robust and reliable EE is considered in the formulation of recommendations. Their key role is to guide the process of incorporating EE into the different sections of the CPG. Each section is associated with one clinical question that the CPG is looking to formulate recommendations about, but not all of them will incorporate EE. The health economist leads the process of obtaining EE, working closely with the members of the GDG, and defining the appropriate way to incorporate EE.

It is recommended that basic training in health economics is provided to all the members of the GDG. This might need to be complemented with additional training if required for adequate interpretation of evidence and/or formulation of recommendations. The GDG needs to be aware that the goal of including economic impact considerations in the formulation of CPGs is to ultimately make recommendations that will yield the largest health gains across the NHS given the available resources. Those interventions that show the greatest ‘efficiency’, that is, those with the greatest benefit per monetary unit invested, are those that should be encouraged. However, evidence of efficiency of one particular intervention under study may not lead to the formulation of a recommendation. Recommendations are formulated by the GDG taking EE into consideration alongside all the non‐EE evidence available to them about the intervention. Similarly, the role of the health economist is that, recommendations are not only based on evidence on the technology's safety and effectiveness, but also its cost‐effectiveness when EE is available. Below, we describe this process for incorporating economic evidence into CPCs in the context of the Spanish NHS.

### 3.2 Stages of the Algorithm for Including EE in CPGs

**Figure 1:**
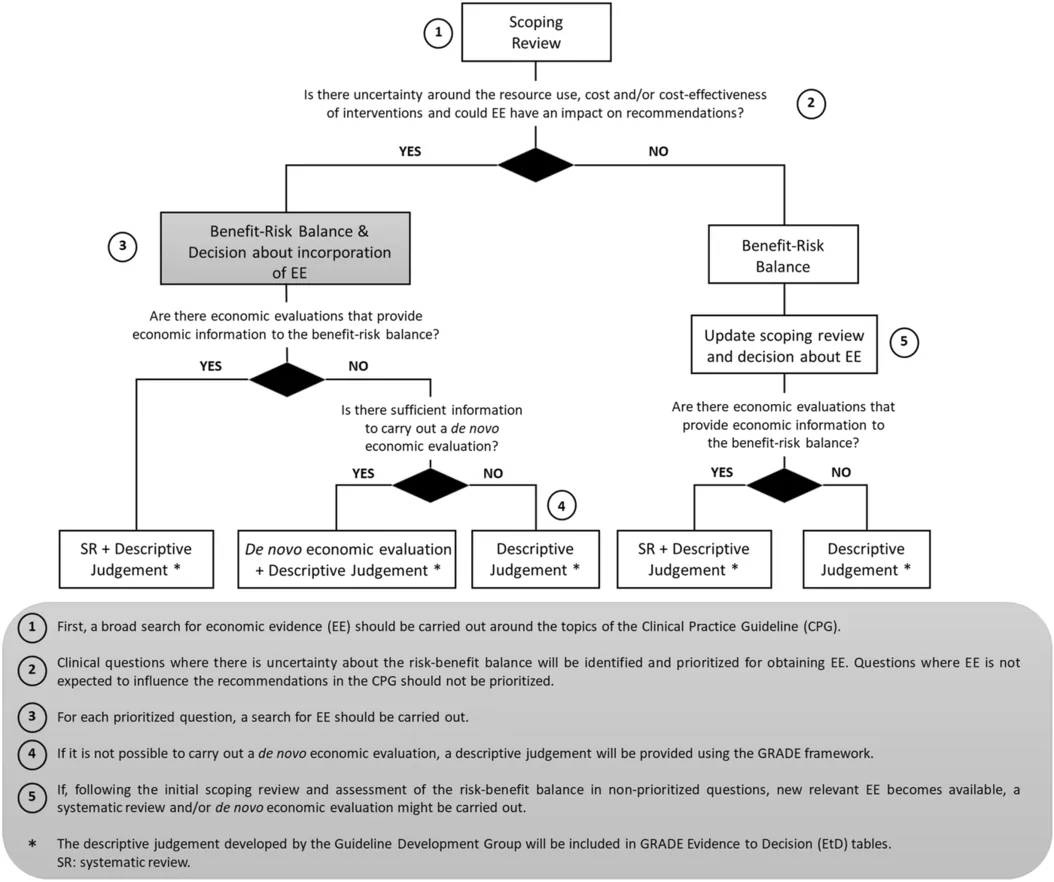
Algorithm for incorporating economic evidence into Clinical Practice Guidelines.

Once the scope and objectives of the CPG have been defined and the GDG has been formed, the clinical questions that will be addressed by the CPG must be defined (see reference [11] for further guidance on how to do this). Once these questions have been defined, the health economist should lead the process of retrieving the EE needed (resource use and costs, and/or cost‐effectiveness) and presenting it to the GDG so that they can use it to formulate recommendations. This process has three main stages (a diagrammatic representation of this process is shown in Figure 1):

1. Prioritise clinical questions in the CPG according to the influence that EE is expected to have on recommendations,
2. Obtain EE for clinical questions that have been prioritised,
3. Present EE to GDG to support the development of recommendations in CPG.

In the following sections, we describe each of the stages outlined above.

#### 3.2.1 Stage 1: Prioritise Clinical Questions in the CPG According to the Influence That EE is Expected to Have on Recommendations

Once clinical questions in the CPG have been defined, it is recommended that a broad search (e.g. a scoping review) of economic aspects around the topic of the guideline is carried out. The goal of this is to, based on the existing EE, prioritise clinical questions (e.g. by ranking them as high vs. low priority) according to whether evidence on use of resources, costs, and cost‐effectiveness can provide valuable information and/or reduce uncertainty (direction and strength) in the formulation of recommendations.

To do so, the GDG may want to consider the following elements:

∘ The influence that EE might have on the recommendations: priority should be given to clinical questions for which the EE is expected to add extra content to the recommendations.
∘ Whether the GDG has sufficient information at this initial stage to be able to determine such influence: priority should be given to clinical questions for which there is certainty that EE will have an important influence on the direction and/or strength of the recommendations.
∘ The availability of resources: when prioritising questions, the GDG should take into account the resources required (technical resources or time) to obtain, combine and/or produce EE.

Then, for high‐priority questions, EE will be retrieved and integrated into a larger body of evidence that will be used by the GDG to inform recommendations. When deciding the number of questions regarded as high priority, the GDG should discuss the availability of time, human or economic resources, taking into account the methodology that will be used for doing so. For low priority questions, EE will in principle not be retrieved or used in formulating recommendations. However, the decision by the GDG about whether EE ought to be retrieved might be revisited if, for example, additional evidence becomes available.

#### 3.2.2 Stage 2: Obtain EE for Clinical Questions That Have Been Prioritised

For each clinical question prioritised in Stage 1, a specific search for EE should be carried out. The methodology for searching, selecting, and synthesising information must be explicitly recorded in the CPG. A SR of economic evaluations should be carried out [13]. If there is sufficient evidence from high‐quality economic evaluations, a SR might be enough to inform recommendations. The following factors may be considered when conducting SRs of economic evaluations to formulate recommendations in a CPG:

∘ The benefit‐risk balance and the available evidence on resource use and the cost of delivering the intervention should be considered alongside each other.
∘ Cost‐effectiveness analysis will be interpreted according to threshold values depending on the specific context (i.e. this threshold will differ between countries).
∘ The certainty of the evidence (i.e. the quality of the economic evaluations considered in light of the assumptions made by the authors, including the risk of bias in the study). GRADE evidence profiles should be used to collect this information.
∘ The transferability and generalisability of the findings to the health context should be evaluated.
∘ The variability and uncertainty of the studies (i.e. whether sensitivity tests show that the findings are robust or sensitive depending on the context parameters or values).

If there is not sufficient information available in the literature to conduct a SR, conducting a *de novo* economic evaluation should be considered as an alternative. It should be an informed decision and carefully weighed with the competing needs for evidence in the CPG. There may be reasons that lead to a decision to not carry out a *de novo* economic evaluation, for example, the unavailability of time or human resources. An alternative possibility to developing EE *de novo*, it is to retrieve evidence on resource use (e.g., days of hospitalisation or visits to hospital emergency rooms) [9] extracted from effectiveness studies and assess them in evidence profiles as critical outcomes variables following the GRADE methodology. Evidence should inform all three economic domains of the GRADE Evidence to Decision (EtD) Framework, that is, (i) how large are the resource requirements?, (ii) what is the certainty of the evidence of resource requirements? and, (iii) are the net benefits worth the incremental cost? [14, 15], and be presented in a narrative manner and discussed with the GDG. Appropriate study designs should that can inform evidence in domains (i) to (iii) should be sought. When neither a SR nor a *de novo* EE is done, this decision should be adequately justified. Sometimes, it may be required that the CPG incorporate a budget impact analysis. This will need to be reported alongside the main results.

#### 3.2.3 Stage 3: Present the EE to GDG to Support the Development of Recommendations in CPG

Following the principles of the GRADE system, economic evidence should be summarised and presented in evidence profiles or summaries of findings (SoF) tables [16]. Currently, the EE‐WG is working to develop guidance on the most appropriate way to present evidence of an economic nature (both on the use of resources as well as costs and/or cost‐effectiveness of the technology), in GRADE evidence profiles and SoF tables. When a SR is carried out, we advise that each economic outcome be presented in a separate table, with a tabulated synthesis of the studies, together with a narrative synthesis of the results and their interpretation.

To structure the information and provide the GDG with transparent, reliable evidence, GRADE Evidence to Decision (EtD) tables can be used [17, 18]. EtD tables have three main elements: formulation of the question, evaluation of the criteria (priority of the problem, magnitude of the desirable and undesirable effects, certainty of the evidence, uncertainty or variability of the assessment and patient preferences regarding to the outcomes of interest, balance of effects, magnitude of resource use and costs, certainty of evidence of required resources, cost‐effectiveness evidence, equity, acceptability, feasibility), and conclusions.

The information on resource use, cost and cost‐effectiveness and the quality of the evidence should be taken together with evidence retrieved on safety, clinical effectiveness, ethical considerations etc. The results of the SR or *de novo* economic evaluation will be included in a table next to a judgement statement. Explicit mention should be made to the approach taken to include EE in the formulation of recommendations.

## 4 Application of the Algorithm to the CPG for the Management of Chronic Primary Pain

### 4.1 The CPG for the Management of Chronic Primary Pain

GuíaSalud's Executive Committee selected the CPP CPG for inclusion in GuíaSalud's CPG Program following a standardised prioritisation process, which takes into account a series of pre‐specified criteria, including incidence and burden of the disease, relevance for the NHS, among others. IACS was commissioned by GuíaSalud to develop the guideline. To that end, a GDG was appointed following the standard procedure for creating a GDG [11]. It included experts from various fields, including one health economist. Following the principles of GRADE‐ADOLOPMENT to guideline production, the GDG decided to evaluate and consider NICE's NG193 Chronic pain (primary and secondary) guideline [19] for adoption, adaptation and generation of *de novo* recommendations. The GDG reviewed the PICO (population, intervention, comparison and outcome) tables for each for clinical question in NG193 and decided that of the questions included there, the new guideline should: (a) not include three of them (electrical physical modalities, acupuncture and manual therapy for CPP), and (b) include a new one (education on neuroscience for CPP). Eleven clinical questions were defined. Then, the application of GRADE‐ADOLOPMENT required to: (i) update all systematic reviews of evidence for clinical questions, (ii) evaluate NICE's approach to evidence rating, and (iii) evaluate NICE's approach to making recommendations. Once all evidence had been gathered in (i) through to (iii), recommendations in NG193 were either adopted as is or adapted by the GDG.

### 4.2 Contents of the CPG

Six of the eleven questions defined required a comparative assessment of the effects of interventions for CPP. These included pharmacological and psychological therapy, as well as education and physical therapy interventions. In NG193, SRs of economic evaluations were conducted for all questions about interventions for CPP [19]. Additionally, two *de novo* economic evaluations were undertook about exercise therapies and acupuncture.

The GDG decided that the algorithm for incorporating EE in CPGs should be used to help with decisions about incorporating EE in the new guideline. To that end, the decisions about which of the six clinical questions to retrieve EE for, and whether that evidence should come from published studies or be generated *de novo* were discussed with the GDG, who made the final decisions. These decisions were made according to, both human and time availability, as well as EE availability for each clinical question, as discussed in Section 3.

### 4.3 Application of the Algorithm in the CPG

**Figure 2:**
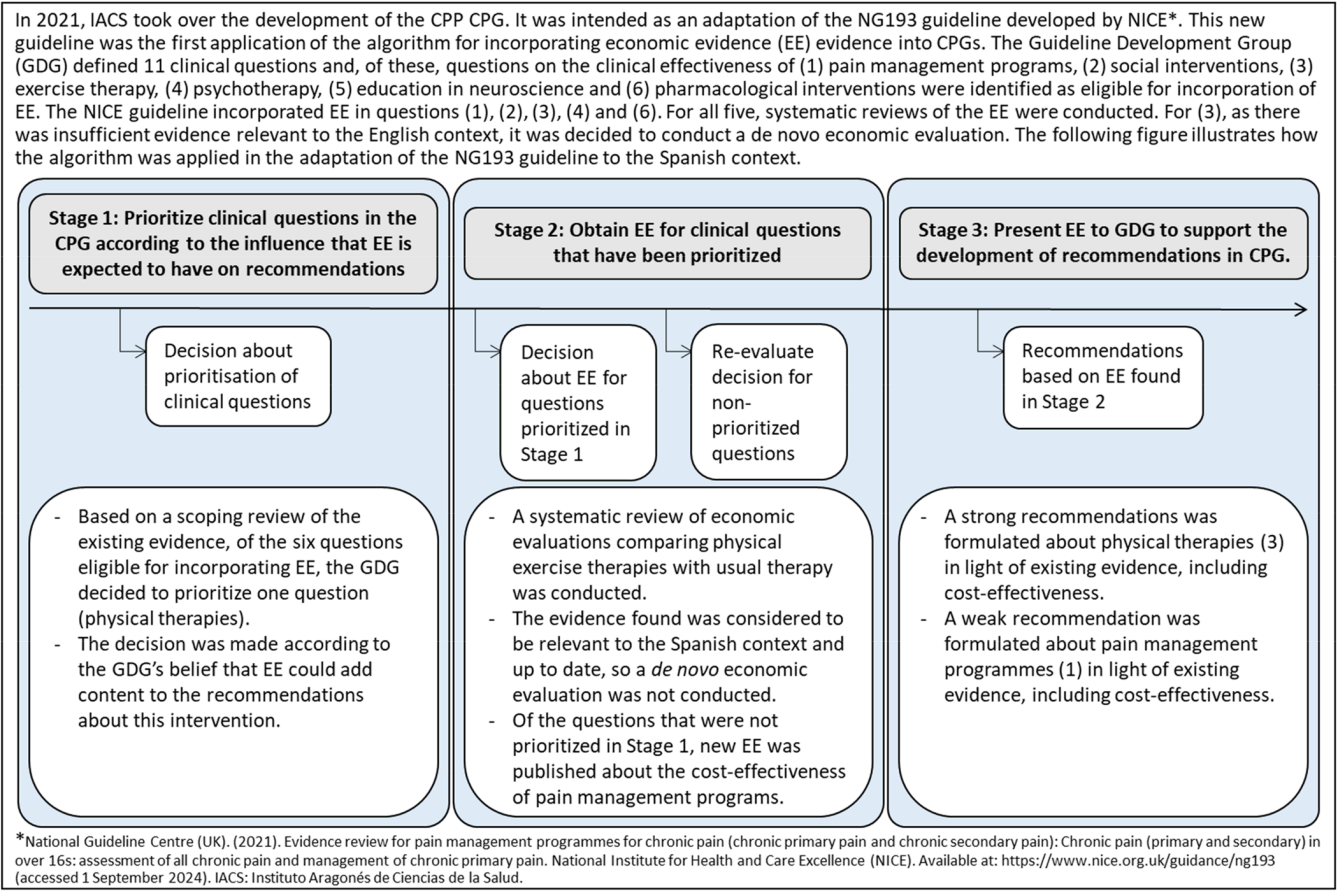
Application of the algorithm for incorporating economic evidence in the clinical practice guideline for the management of chronic primary pain (CPP CPG).

Once all questions had been defined, the algorithm was used to determine which of the six questions about interventions would require an assessment of the economic aspects of the intervention under study (the process is illustrated in Figure 2). The decision made by the GDG was to prioritise the question about physical exercise therapies for CPP. The application of GRADE‐ADOLOPMENT required that for this question: (1) studies included in NICE's SR of economic evaluations were reviewed, (2) the SR in NG193 was updated (including studies published after NG193 as well as *de novo* evidence generated in NG193), and (3) recommendations in NG193 about exercise therapies were either adopted or adapted. In addition to the two studies included in NG193, one of which was conducted in the Spanish context, the CPG technical team identified two other economic evaluations, including the one conducted *de novo* in NG193. For the other five questions, the decision made was that EE would not be obtained or used in the formulation of recommendations. However, as the new guideline progressed, new evidence on the cost‐effectiveness of pain management programmes for CPP was published. The GDG determined that these studies were relevant to the clinical question as they had been conducted in the Spanish context, and decided to include them.

For the two clinical questions for which EE was collected, conducting de novo economic evaluations was not deemed pertinent by the GDG, due to the availability of EE from high‐quality economic evaluations conducted in the Spanish NHS, and the lack of reliable data from the Spanish context on resource use and unit costs for the interventions. For both clinical questions, once all relevant economic studies had been identified, evidence were presented to the GDG for their consideration. In particular, the evidence informed all three economic criteria of the GRADE Evidence to Decision (EtD) Framework [14, 15]. When discussing resource use estimates in domain (i), “how large are the resource requirements?”, the GDG were presented not only with physical therapy cost magnitude estimates, but also whether estimates were robust to sensitivity analysis. In domain (ii), “what is the certainty of the evidence of resource requirements?”, the limitations of the studies identified and how they affected the certainty of the evidence were presented. In domain (iii), “are the net benefits worth the incremental cost?” of the EtD table, cost‐effectiveness ratios were presented alongside the cost‐effectiveness threshold used in the Spanish setting and sensitivity analyses (when available). All cost estimates from studies identified were converted to euros of 2023.

In both prioritised questions, reviews of both clinical and economic aspects of the interventions led to favourable recommendations about the interventions. In the case of the physical exercise question, following the GRADE‐adolopment methodology, a favourable recommendation for supervised group exercise programmes in patients with CPP was adapted (from weak to strong). A second recommendation to stay physically active was adapted to recognise that that the therapy should be tailored to the patient's needs and preferences and be designed to maximise adherence. With regard to the pain management programmes for CPP, a good clinical practice recommendation was generated *de novo*.

## 5 Discussion

The assessment of evidence about resource use, cost, and cost‐effectiveness of an intervention are critical elements in the process of CPG development and formulation of recommendations, especially when the desirable and undesirable clinical effects are balanced. The lack of clear guidance on how such evidence should be incorporated has led to practice variation. Our algorithm can help articulate decisions about evidence needs, and ensure the process of collecting and reviewing EE to inform recommendations in CPG is made in an agile, rigorous and reproducible manner. This paper illustrates how the algorithm for incorporating EE into CPGs could be used in the development of a CPG.

The algorithm can help developers make pragmatic decisions about where evidence is most needed and target resources accordingly. The first stage requires identifying and prioritising areas where evidence is needed. In situations where no evidence on the benefit of interventions is available or where the benefit‐risk ratio is definitely positive or negative, the decision might be that EE will have little influence on the recommendation for or against the intervention and therefore is not going to be obtained. Oftentimes, there will be uncertainty around the benefit‐risk ratio of one or more of the clinical interventions under study in the CPG. In those cases, EE could help add extra content to the recommendations formulated. The second stage is to make a decision about the type of evidence needed. That is, whether a SR of existing evidence and/or *de novo* evidence generation is needed. The third step requires using EE to formulate recommendations. The proposed approach recognises that, often, the decision about whether EE should be obtained for formulating evidence‐based recommendations in CPGs might to be revisited as the process of developing the guideline progresses. For that reason, this algorithm is not intended to be rigid. Developers can and should adapt the strategy to evidence retrieval (systematic review or generation) as further evidence becomes available and experience is gained through the CPG development process. In addition to facilitating agile and dynamic decision‐making, the algorithm promotes the transparency and reproducibility of methods used in the formulation of recommendations and CPG development. However, the use of the algorithm is constrained by differences in methodological capacity across organisations and the requirement for health economics expertise within guideline development teams.

Some other known issues with the development of CPGs, including how to deal with low external validity of studies in SRs, that explain to a large degree the still limited role that EE plays in the development of CPGs, have not been addressed in this study. In fact, the proposed algorithm should be used alongside existing tools for reviewing and generating de novo EE for CPG. For instance, the ISPOR‐AMPC‐NPC tool [20] could be used when evaluating the relevance and credibility of existing nonexperimental evidence. Future work will need to consider other issues, such as the lack of guidance on how to assess the transferability and generalisability of EE, which constitute important barriers for the development of recommendations [21]. As real‐world data becomes more widely available for HTA and CPG development, we anticipate that the algorithm will need to be adapted to consider real‐world evidence when formulating recommendations, especially in those settings like personalised medicine where experimental evidence is insufficient or inadequate.

## Author Contributions

**Celia Muñoz:** conceptualization, cethodology, investigation, validation, writing – original draft, writing – review and editing. **Patricia Gavín‐Benavent:** conceptualization, writing – review and editing, methodology. **Silvia Moler‐Zapata:** conceptualization, writing – review and editing, writing – original draft, methodology, validation, investigation. **Lucía Prieto Remón:** writing – original draft. **María Bono Vega:** conceptualization, writing – review and editing, writing – original draft. **Soledad Isern de Val:** conceptualization, writing – original draft, writing – review and editing, supervision, project administration, methodology, investigation, validation.

## Ethics Statement

The authors have nothing to report.

## Consent

The authors have nothing to report.

## Conflicts of Interest

The authors declare no conflicts of interest.

## Data Availability

Data sharing is not applicable to this article as no new data were created or analysed in this study.

## References

[1] M. F. Drummond , et al., Methods for the Economic Evaluation of Health Care Programmes (Oxford University Press, 2015).

[2] Asociación de Economía de la Salud . Posición de la asociación de economía de la salud en relación a la necesidad de un mayor uso de la evaluación económica en las decisiones que afectan a la financiación pública de las prestaciones y tecnologías en el sistema nacional de salud; (2008), https://www.aes.es/Publicaciones/AESEE2.pdf.

[3] Ministerio de Sanidad, Consumo y Bienestar Social ., Memoria de Actividades 2006‐2016. Diez Años Cooperando En Evaluación De Tecnologías Sanitarias (Madrid: Ministerio de Sanidad, Consumo y Bienestar Social. Red Española de Agencias de Evaluación de Tecnologías Sanitarias y Prestaciones del Sistema Nacional de Salud (RedETS), (2019), https://redets.sanidad.gob.es/documentos/MEMORIA_DIEZ_ANNOS_03_Final_Revisado.pdf.

[4] Ministerio de Sanidad, Consumo y Bienestar Social . Plan Estratégico RedETS 2022–2025. (2020), https://redets.sanidad.gob.es/Presentacion_PlanEstrategico/Archivos/PLAN_ESTRATEGICO_RedETS.pdf.

[5] G. H. Guyatt , A. D. Oxman , G. E. Vist , et al., “GRADE: An Emerging Consensus on Rating Quality of Evidence and Strength of Recommendations,” BMJ 336, no. 7650 (2008): 924–926, 10.1136/bmj.39489.470347.AD. 18436948 PMC2335261

[6] J. Andrews , G. Guyatt , A. D. Oxman , et al., “GRADE Guidelines: 14. Going From Evidence to Recommendations: The Significance and Presentation of Recommendations,” Journal of Clinical Epidemiology 66, no. 7 (2013): 719–725, 10.1016/j.jclinepi.2012.03.013. 23312392

[7] J. C. Andrews , H. J. Schünemann , A. D. Oxman , et al., “GRADE Guidelines: 15. Going From Evidence to Recommendation‐Determinants of a Recommendation's Direction and Strength,” Journal of Clinical Epidemiology 66, no. 7 (2013): 726–735, 10.1016/j.jclinepi.2013.02.003. 23570745

[8] H. Balshem , M. Helfand , H. J. Schünemann , et al. (“Grade Guidelines: 3. Rating the Quality of Evidence,” Journal of Clinical Epidemiology 64, no. 4 (2011): 401–406, 10.1016/j.jclinepi.2010.07.015. 21208779

[9] M. Brunetti , I. Shemilt , S. Pregno , et al., “GRADE Guidelines: 10. Considering Resource Use and Rating the Quality of Economic Evidence,” Journal of Clinical Epidemiology 66, no. 2 (2013): 140–150, 10.1016/j.jclinepi.2012.04.012. 22863410

[10] F. Xie , I. Shemilt , L. Vale , et al., “GRADE Guidance 23: Considering Cost‐Effectiveness Evidence in Moving From Evidence to Health‐Related Recommendations,” Journal of Clinical Epidemiology 162 (2023): 135–144, 10.1016/j.jclinepi.2023.08.001. 37597696

[11] Grupo de trabajo para la actualización del Manual de Elaboración de GPC ., Elaboración de Guías de Práctica Clínica en el Sistema Nacional de Salud. Actualización del Manual Metodológico [Internet] (Ministerio de Sanidad, Servicios Sociales e Igualdad; Zaragoza: Instituto Aragonés de Ciencias de la Salud (IACS), (2016), http://portal.guiasalud.es/emanuales/elaboracion_2/?capitulo.

[12] National Institute for Health and Care Excellence (NICE) ., “Incorporating Economic Evaluation.” Developing NICE Guidelines: The Manual (NICE, 2014), https://www.nice.org.uk/process/pmg20/chapter/incorporating-economic-evaluation.

[13] G. A. P. G. Van Mastrigt , M. Hiligsmann , J. J. C. Arts , et al., “How to Prepare a Systematic Review of Economic Evaluations for Informing Evidence‐Based Healthcare Decisions: A Five‐Step Approach (Part 1/3),” Expert Review of Pharmacoeconomics and Outcomes Research 16, no. 6 (2016): 689–704. 27805469 10.1080/14737167.2016.1246960

[14] P. Alonso‐Coello , A. D. Oxman , J. Moberg , et al., “GRADE Evidence to Decision (EtD) Frameworks: A Systematic and Transparent Approach to Making Well Informed Healthcare Choices. 2: Clinical Practice Guidelines,” BMJ 353 (2016): i2089, 10.1136/bmj.i2089. 27365494

[15] P. Alonso‐Coello , H. J. Schünemann , J. Moberg , et al., “GRADE Evidence to Decision (EtD) Frameworks: A Systematic and Transparent Approach to Making Well Informed Healthcare Choices. 1: Introduction,” BMJ 353 (2016): 2016, 10.1136/bmj.i2016. 27353417

[16] G. Guyatt , A. D. Oxman , E. A. Akl , et al., “GRADE Guidelines: 1. Introduction‐Grade Evidence Profiles and Summary of Findings Tables,” Journal of Clinical Epidemiology 64, no. 4 (2011): 383–394, 10.1016/j.jclinepi.2010.04.026. 21195583

[17] H. J. Schünemann , R. Mustafa , J. Brozek , et al., “GRADE Guidelines: 16. GRADE Evidence to Decision Frameworks for Tests in Clinical Practice and Public Health,” Journal of Clinical Epidemiology 76 (2016): 89–98, 10.1016/j.jclinepi.2016.01.032. 26931285

[18] J. Moberg , A. D. Oxman , S. Rosenbaum , et al., “The GRADE Evidence to Decision (EtD) Framework for Health System and Public Health Decisions,” Health Research Policy and Systems 16, no. 1 (2018): 45, 10.1186/s12961-018-0320-2. 29843743 PMC5975536

[19] National Guideline Centre (UK) ., Evidence Review for Pain Management Programmes for Chronic Pain (Chronic Primary Pain and Chronic Secondary Pain): Chronic Pain (Primary and Secondary) in Over 16s: Assessment of all Chronic Pain and Management of Chronic Primary Pain (National Institute for Health and Care Excellence (NICE), 2021), https://www.nice.org.uk/guidance/ng193. 33939361

[20] J. Jaime Caro , D. M. Eddy , H. Kan , et al., “Questionnaire to Assess Relevance and Credibility of Modeling Studies for Informing Health Care Decision Making: An ISPOR‐AMCP‐NPC Good Practice Task Force Report,” Value in Health 17, no. 2 (2014): 174–182, 10.1016/j.jval.2014.01.003. 24636375

[21] A. Weise , R. B. Büchter , D. Pieper , and T. Mathes , “Assessing Transferability in Systematic Reviews of Health Economic Evaluations–A Review of Methodological Guidance,” BMC Medical Research Methodology 22, no. 1 (2022): 52, 10.1186/s12874-022-01536-6. 35184733 PMC8858549

